# Low Density Lipoprotein Receptor Variants in the Beta-Propeller Subdomain and Their Functional Impact

**DOI:** 10.3389/fgene.2020.00691

**Published:** 2020-06-30

**Authors:** Lucie Dušková, Lucie Nohelová, Tomáš Loja, Jana Fialová, Petra Zapletalová, Kamila Réblová, Lukáš Tichý, Tomáš Freiberger, Lenka Fajkusová

**Affiliations:** ^1^Centre of Molecular Biology and Gene Therapy, University Hospital Brno, Brno, Czechia; ^2^Central European Institute of Technology, Masaryk University, Brno, Czechia; ^3^Centre for Cardiovascular Surgery and Transplantation, Brno, Czechia; ^4^Medical Faculty, Masaryk University, Brno, Czechia; ^5^Laboratory of Functional Genomics and Proteomics, National Centre for Biomolecular Research (NCBR), Faculty of Science, Masaryk University, Brno, Czechia

**Keywords:** low density lipoprotein receptor, live cell imaging microscopy, flow cytometry, functional analysis, ER stress

## Abstract

**Background:** Pathogenic variants in the low density lipoprotein receptor gene are associated with familial hypercholesterolemia. Some of these variants can result in incorrect folding of the LDLR protein, which is then accumulated inside the cell and cannot fulfill its function to internalize LDL particles. We analyzed the functional impact of 10 LDLR variants localized in the beta-propeller of epidermal growth factor precursor homology domain. The experimental part of the work was complemented by a structural analysis on the basis of 3D LDLR protein structure.

**Methods:** T-Rex Chinese hamster ovary cells transfected with the human LDLR gene were used for live cell imaging microscopy, flow cytometry, and qRT-PCR analysis.

**Results:** Our results showed that the analyzed LDLR protein variants can be divided into three groups. (1) The variants buried inside the 3D protein structure expressing proteins accumulated in the endoplasmic reticulum (ER) with no or reduced plasma membrane localization and LDL particle internalization, and associated with an increased gene expression of ER-resident chaperones. (2) The variants localized on the surface of 3D protein structure with slightly reduced LDLR plasma membrane localization and LDL particle internalization, and associated with no increased mRNA level of ER-resident chaperones. (3) The variants localized on the surface of the 3D protein structure but expressing proteins with cell responses similar to the group 1.

**Conclusion:** All analyzed LDLR variants have been evaluated as pathogenic but with different effects on protein localization and function, and expression of genes associated with ER stress.

## Introduction

Familial hypercholesterolemia (FH) is characterized by elevated low-density lipoprotein cholesterol levels, which lead to accelerated atherosclerosis and premature coronary heart disease. FH frequency in most populations is estimated to be 1:200–250 (Benn et al., 2012; Sjouke et al., 2015). FH is an autosomal dominant disease associated with pathogenic variants in the low density lipoprotein receptor gene (LDLR), the apolipoprotein B gene (APOB), or the proprotein convertase subtilisin/kexin type 9 gene (PCSK9) (Brown and Goldstein, 1986; Rader et al., 2003).

The LDLR gene encodes a protein of 860 amino acids comprising a 21 amino acid signal sequence at the N-terminus. This sequence is excised during protein translocation into the endoplasmic reticulum (ER) (Strom et al., 2011). LDLR is synthesized on ER membrane-bound ribosomes, folded and partially glycosylated within the ER lumen and finally matured in the Golgi complex, where glycosylation is completed. Approximately 45 min after synthesis, LDLR appears on the cell surface and mediates LDL particle uptake by receptor mediated endocytosis. The LDL particle is then released in the endosome, and the protein recycles, i.e., it returns to the cell membrane (Brown et al., 1997). The complex function of the LDL receptor is ensured by its specific functional domains: an N-terminal ligand-binding domain composed of seven ligand binding repeats (R1-R7); an epidermal growth factor precursor homology domain composed of two epidermal growth factor (EGF)-like modules (A and B), a six-bladed beta-propeller, and a third EGF-like module (C) connected to an O-linked sugar domain followed by a transmembrane domain and C-terminal cytoplasmic tail (Hobbs et al., 1990).

LDLR variants have different effects on the protein expression, maturation, localization, and function. If LDLR reaches its native conformation, it is released from ER through the protein secretory pathway. Accumulation of misfolded proteins in the ER lumen can trigger ER stress and unfolded protein response (UPR), which activates an adaptive response to restore ER homeostasis by (1) lowering protein synthesis and translocation into ER, (2) increasing molecular chaperone expression to enhance protein folding capacity in ER, and (3) inducing apoptosis when UPR fails to re-establish ER homeostasis (Ron and Walter, 2007; Minamino et al., 2010; Hetz, 2012; Hetz et al., 2015).

In this study, we examined the impact of 10 missense LDLR sequence variants localized in the beta-propeller subdomain on the protein localization and function. Further, we investigated mRNA levels of ER-resident chaperones CALR (calreticulin), HSPA5 (heat-shock 70-kD protein 5), HSP90B1 (heat-shock 90-kD protein beta 1); and ER-resident protein HERPUD1 (homocysteine- and ER stress-inducible protein ubiquitin-like domain-containing 1). CALR is responsible for folding of synthesized glycoproteins and quality control in the calnexin/calreticulin cycle (Hebert and Molinari, 2007; Michalak et al., 2009). HSPA5 interacts with the luminal domain of three ER transmembrane proteins (the activating transcription factor 6, the eukaryotic translation initiation factor 2-alpha kinase 3, and the endoplasmic reticulum-to-nucleus signaling 1), which act as proximal UPR sensors under non-stressed conditions. With the accumulation of unfolded and misfolded proteins, HSPA5 binds to these proteins and this activates the mentioned UPR sensors (Ye et al., 2000; Back et al., 2005). HSP90B1 participates in protein folding, interacts with other components of the ER protein folding machinery, stores ER calcium, and assists in targeting misfolded proteins for the ER associated degradation (ERAD) (Eletto et al., 2010; Marzec et al., 2012). HERPUD1 plays a protective role in ER stress-induced apoptosis (Chan et al., 2004). HERPUD1 interaction with other ERAD components, proteasomes, and ubiquitinated substrates (Schulze et al., 2005) makes it a good candidate to coordinate UPR and ERAD processes, which are interdependent (Travers et al., 2000).

The experimental results were complemented by *in silico* variant analysis on the basis of the human 3D LDLR protein structure.

## Materials and Methods

### Selection of LDLR Variants

Ten LDLR variants located in the beta-propeller of epidermal growth factor precursor homology domain were investigated. Six of them were found in Czech FH patients and also described in other FH populations – p.Glu408Val, c.1223A > T (exon 9); p.Arg416Trp, c.1246C > T (exon 9); p.Val429Met, c.1285G > A (exon 9); p.Gly478Arg, c.1432G > A (exon 9); p.His583Arg, c.1748A > G (exon 12); and p.Ser610Cys, c.1829C > G (exon 12) (Tichy et al., 2012; Vrablik et al., 2017). The variant p.Tyr532Cys, c.1595A > G (exon 11) was identified only in one Czech FH patient (Tichy et al., 2017). The other three variants were selected either on the basis of 3D protein structural analysis [p.Leu555Pro, c.1664T > C (exon 11); p.His583Tyr, 1747C > T (exon 12)] or on the basis of published studies [p.Gly565Val, c.1694G > T (exon 11)] as a reference for variants associated with complete ER protein retention (Sorensen et al., 2006). The LRG_274 reference sequence was used for nomenclature of the LDLR sequence variants.

### Site-Directed Mutagenesis and Transfection

The pcDNA4-LDLR-linker-EYFP plasmid (kindly provided from MA Kulseth, Norway) was used for T-Rex CHO (Chinese hamster ovary) cell line transfections (Life Technologies). The T-Rex system allows induction of a transfected gene expression by tetracycline. The EYFP (Enhanced Yellow Fluorescent Protein) tag, inserted at the C-terminus of LDLR cDNA and separated by a 10 amino acid linker, was used to visualize the protein in cells. The EYFP tag does not affect the LDLR biosynthesis pathway (Sorensen et al., 2006). The LDLR sequence variants were created by a site-directed mutagenesis (QuickChange Mutagenesis Lightning kit, Agilent Technologies) and verified by DNA sequencing. T-Rex CHO cells were transfected with plasmids carrying wild type (wt) or mutated LDLR cDNA by lipid-mediated transfection (Lipofectamine 2000, Life Technologies). CHO cell lines were grown in Ham′s F12 medium supplemented with fetal bovine serum (10%, MP Biomedicals), L-glutamine (2 mM, Sigma-Aldrich), and blasticidin (10 μg/ml, Invitrogen) in 37°C and 5% CO_2_. Stable CHO cell lines were generated using zeocin selection (600 μg/ml, Invitrogen).

### Live Cell Imaging Microscopy of LDLR, ER, and Internalized LDL Particles

Live cell imaging microscopy was used to study LDLR localization on the plasma membrane and ER, and LDL particle uptake. ER was visualized using CellLight^®^ ER-RFP, BacMam 2.0 Reagent (Life Technologies), which is a fusion construct of ER signal sequence of calreticulin and KDEL (ER retention signal) with a Red Fluorescent Protein (RFP) tag packaged in the insect Baculovirus. T-Rex CHO cells were seeded (5 × 103) into 8-well chambers for live cell imaging (Cellvis C8-1,5H-N, Bio-Port) and transduced with 2 μl of BacMam 2.0 Reagent. Twenty-four hours after transduction, LDLR expression was induced by adding tetracycline (1 μg/ml, Sigma-Aldrich) for 24 h. The nucleus of the cells was labeled with Hoechst 33342 (1:2000, Thermo Fisher Scientific) for 45 min at 37°C. Finally, the cells were washed with 1× PBS (Phosphate Buffered Saline) and observed in Live Imaging Solution (Life Technologies) with Live Imaging Antifade (1:100, Life Technologies) on Zeiss LSM 880 laser scanning confocal microscope at 37°C and 5% CO_2_. The images were analyzed by the ZEN software. Samples for each LDLR protein variant were prepared at least three times.

To study the function of the LDL receptor, LDL particles fluorescently labeled with pHRodo-Red (0.5 mg/ml, Life Technologies) were added to the cells expressing LDLR after 24 h of tetracycline induction and incubated for 40 min. After the incubation time, the cells were prepared for live imaging microscopy and analyzed as described above. The pHRodo-Red label gives fluorescence only in acidic pH and thus reflects LDL particles internalized by the cell and present in endosomes or lysosomes.

### Flow Cytometry of LDLR Cell Surface Expression and LDL Particle Internalization

Flow cytometry was used for determination of LDLR plasma membrane localization and its ability to internalize LDL particles. T-Rex CHO cells were seeded into 12-well plates (Nunc, Life Technologies), LDLR expression was induced by adding tetracycline (1 μg/ml, Sigma-Aldrich) for 24 h. To determine LDLR plasma membrane localization, monoclonal anti-hLDLR APC-conjugated Mouse IgG1 antibody (1μl/105 cells, R&D Systems) was added to the cells and incubated overnight at 37°C and 5% CO_2_. For determination of LDL particle internalization, LDL particles fluorescently labeled with pHRodo-Red (0.5 mg/ml, Life Technologies) were added to the cells and incubated for 40 min. After the incubation times, the cells were detached using Accutase (Life Technologies), washed with 1× PBS and suspended in FACS buffer for flow cytometric analysis on BD FACS Verse (BD Systems). Sytox blue staining (0.2 μM, Life Technologies) was used to separate dead and live cells. For each sample, 104 of single cells were analyzed. The median of APC or pHRodo-Red fluorescence intensity obtained from cells expressing a LDLR variant was compared to that obtained from wt LDLR. For each LDLR variant, the results are presented as a mean of triplicate experiments ± standard deviation (S.D.) compared with the wt LDLR, which represents 100%. Statistical significance was determined using the Dunnett’s test with 95% confidence interval (*p* < 0.05) in the GraphPad Prism statistic software.

### Quantitative mRNA Analysis of ER-Resident Proteins

The mRNA levels of selected genes were analyzed by quantitative RT-PCR (qRT-PCR) in T-Rex CHO cell lines stably transfected with wt LDLR or mutant LDLR. The cells were harvested 24 h after tetracycline induction (1 μg/ml, Sigma-Aldrich) into RNA Protect Cell Reagent (Qiagen). The total RNA was isolated using RNeasy Plus Mini Kit with on-column gDNA removal (Qiagen) and additional DNaseI treatment. RNA integrity was measured with Agilent 2100 Bioanalyzer (Agilent Technologies). Reverse transcription (RT) was carried out with 2 μg RNA using High Capacity RNA to cDNA Kit (Applied Biosytems). The qRT-PCR was performed in 96 well MicroAmp Fast Optical Reaction Plates (Applied Biosystems) with 20× TaqMan Gene Expression Assays [Cg04421473_g1 (Calr), Cg04423734_g1 (Hspa5), Cg04548386_g1 (Hsp90b1), Cg04495044_m1 (Herpud1), Cg04424038_gH (Gapdh); Applied Biosystems] and 2× TaqMan Fast Advanced Master Mix (Applied Biosystems) on 7900HT Fast Real-Time PCR system (Applied Biosystems). The results of expression of target genes were normalized against Gapdh as a reference control with verified stable gene expression, mRNA from wt LDLR transfected cell line served as a calibrator. All qRT-PCR measurements were run in triplicates. The gene expression measurements from three biological replicates were performed and the results are presented as mean ± S.D. The levels of significance were determined using the Dunnett’s test with 95% confidence interval (*p* < 0.05) in the GraphPad Prism statistic software.

### Structural Analysis of LDLR Variants

We analyzed the structural effect of 10 variants based on the LDLR human X-ray structure determined at pH = 5.3 (3.7 Å resolution), which should represent conformation adopted in endosomes (PDB code: 1N7D) (Rudenko et al., 2002). In this conformation, ligand binding repeats R4 and R5 interact with beta-propeller while on the membrane at neutral pH, the repeats bind various lipoprotein particles. Similarly to the previous study (Reblova et al., 2015), we analyzed the wt amino acids (AAs) side chain contacts, i.e., direct H-bonds, salt bridges, and stacking interactions using the VMD program (Humphrey et al., 1996). In addition, we measured the buriedness of the wt AAs in the protein structure. The residues’ solvent accessibility in the protein structure was calculated using the STRIDE program (Frishman and Argos, 1995) and divided by the total surface area of the residue (Chothia, 1976). This value corresponds to the relative accessible surface area (RSA). A residue was considered buried if RSA is ≤10%. Replacing a buried AA is more likely to be associated with structural defects especially when volume, charge, and polarity change upon a sequence substitution, and thus we measured these parameters for buried residues. Volume change upon a missense variant was calculated (Zamyatnin, 1972), a change ≥20 Å3 associated with the large to small substitution was considered destabilizing. A charge change upon a variant was considered between charged and uncharged AAs and a polarity change was considered between nonpolar (Leu, Ile, Phe, Trp, Cys, Met, Val, Tyr), polar (Pro, Ala, Thr, Gly, Ser), and very polar (His, Arg, Gln, Lys, Asn, Glu, Asp) AAs. Further, we detected the replacement of wt proline and glycine residues in turns where they are key structural factors. In addition, substituting any AA to proline in alpha-helical and beta-sheet structures was considered destabilizing (Li et al., 1996).

## Results

**FIGURE 1:**
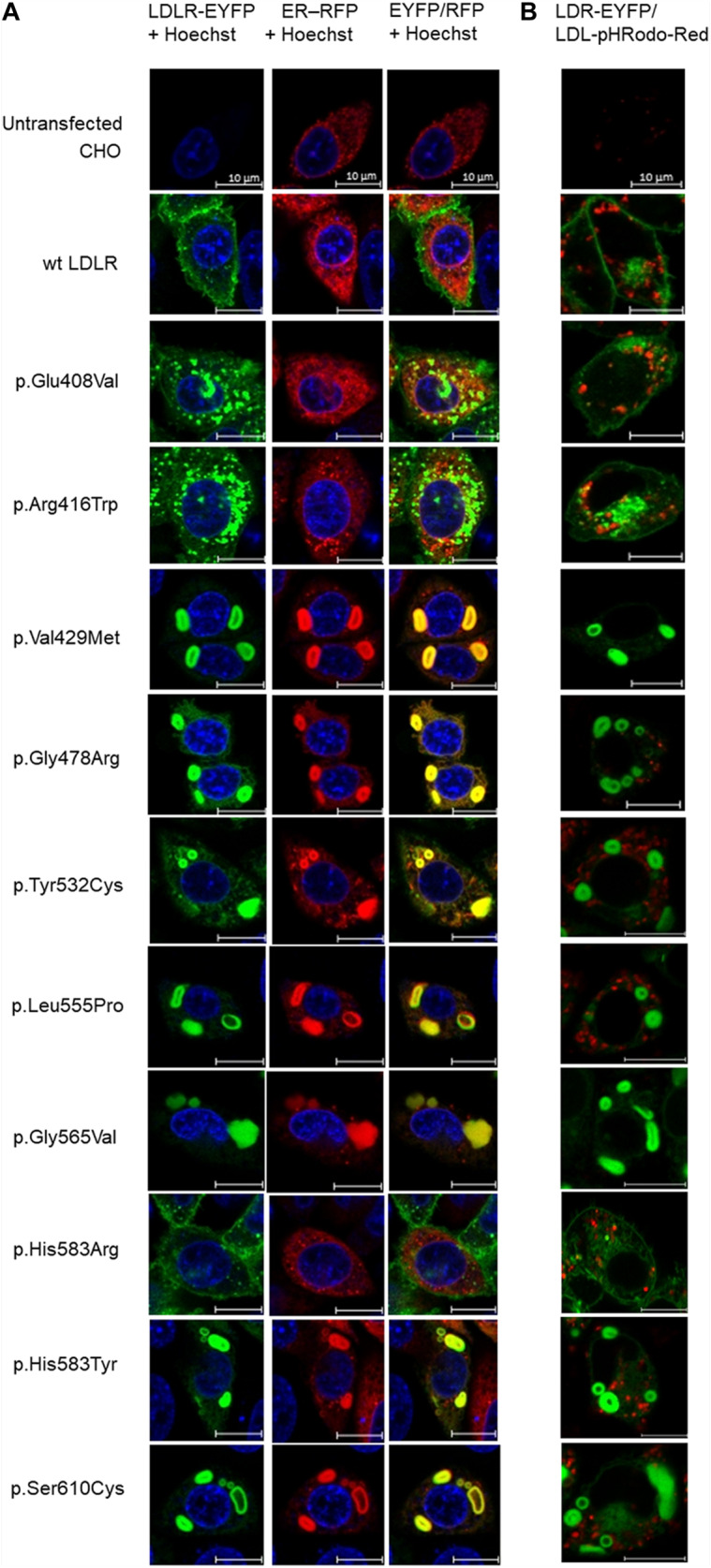
Live cell imaging microscopy of LDLR and ER **(A)**. LDLR was visualized via EYFP (green), ER via RFP (red). The LDLR presence on ER was evaluated by EYFP/RFP fluorescence overlay (yellow). The cell nucleus was stained with Hoechst 33342 (blue). Live cell imaging microscopy of LDLR and internalized LDL particles **(B)**. LDLR was visualized via EYFP (green), LDL particles via pHRodo-Red (red). Representative cells are presented. Scale bar represents 10 μm.

**FIGURE 2:**
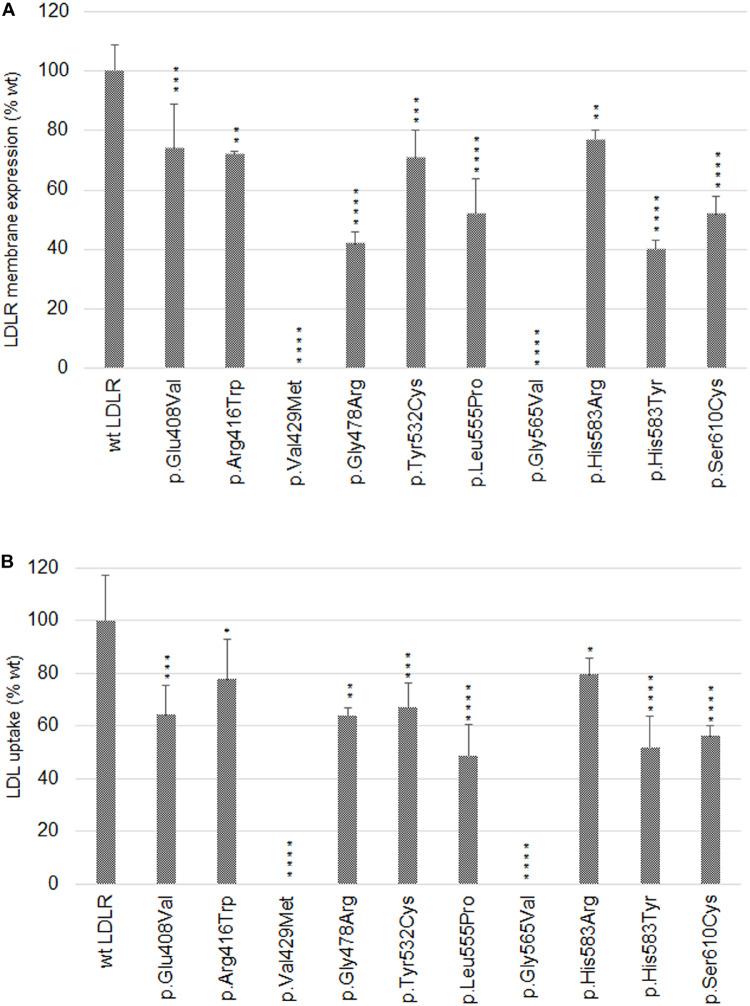
Graphic evaluation of flow cytometry analyses of LDLR plasma membrane expression **(A)** and LDL particle internalization **(B)**. The values represent the mean of three independent experiments; error bars ± S.D. Statistical significance was determined using Dunnett’s test. The statistical significance degree of difference: *p < 0.05, **p < 0.01, ***p < 0.001, ****p < 0.0001.

**FIGURE 3:**
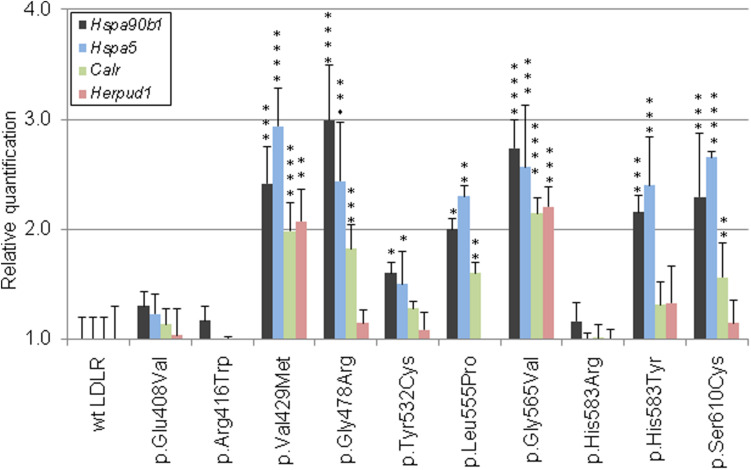
Graphic evaluations of quantitative mRNA analyses of ER-resident proteins. The mRNA levels of Hsp90b1, Hspa5, Calr, and Herpud1 were determined by qRT-PCR. The values represent the mean of three independent experiments performed in triplicates; error bars ± S.D. The statistical significance was determined by the Dunnett’s test. The statistical significance degree of difference: *p < 0.05, **p < 0.01, ***p < 0.001, ****p < 0.0001.

**FIGURE 4:**
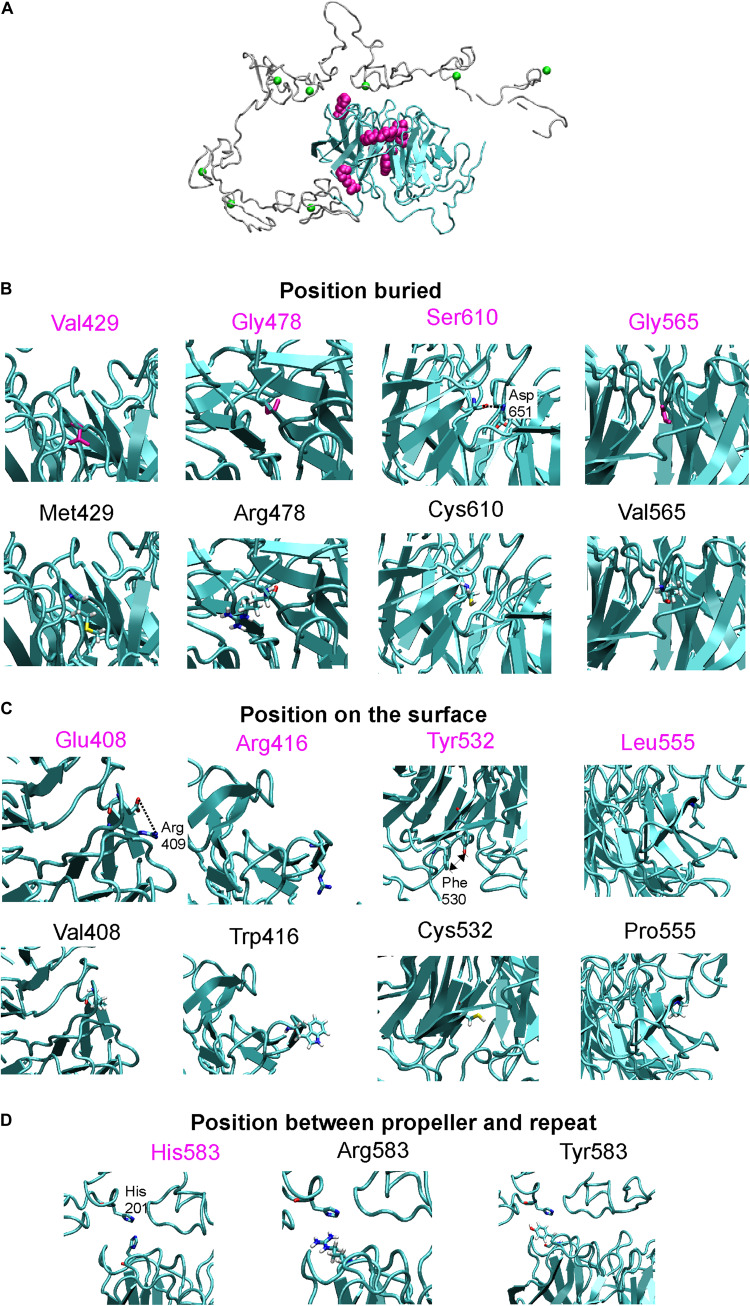
**(A)** X-ray structure of LDLR with highlighted beta-propeller (in cyan) and studied amino acid positions (in magenta surface representation), Ca^2+^ ions bound in repeats are in green. Detailed view on wt and mutant structures which were divided according to amino acid positions into positions buried inside the protein **(B)**, positions on the protein surface **(C)**, and positions between the beta-propeller and the R5 repeat **(D)**. These views show interaction between studied amino acids and surroundings, note salt bridge between Glu408-Arg409, H-bonding between Ser610-Asp651, or stacking between Tyr532 and Phe530. Close contact between His583 from propeller and His201 from repeat 5 is also shown.

Analyzed LDLR variants were evaluated for their occurrence in Human Gene Mutation Database (HGMD)^1^, ClinVar^2^, and Leiden Open Variation Database (LOVD)^3^. Using live cell imaging microscopy and flow cytometry, qualitative and quantitative analyses were performed evaluating selected variants in terms of protein localization (Figures 1A, 2A) and its ability to internalize LDL particles (Figures 1B, 2B). Untransfected T-Rex CHO cells served as a negative control in microscopic and flow cytometric analyses. Further, mRNA analysis of selected genes encoding ER-resident proteins were performed for analysis of possible association between the variant and ER stress activation (Figure 3). The experimental part of the work was complemented by a structural analysis of particular variants on the basis of 3D LDLR protein structure (Figure 4 and Table 1).

**TABLE 1:** Summary of experimental and structural analysis results.

Protein variant	Contact via side chain	Buriedness (RSA)	Change of			Pro/Gly change	LCIM	FC (loc., % of wt)	FC (LDL int., % of wt)	Increased chaperone expression	Group
			Charge	Polarity	Volume						
wt	NA	NA	NA	NA	NA	NA	Membrane + ER	100 ± 9	100 ± 17	No	–
p.Glu408Val		26%					Membrane + ER	74 ± 15	64 ± 11	No	2
p.Arg416Trp		54%					Membrane + ER	72 ± 1	78 ± 15	No	2
p.Val429Met		6%					ER^a^	0	0	Yes	1
p.Gly478Arg		0%					ER^a^	42 ± 4	64 ± 3	Yes	1
p.Tyr532Cys		14%					ER^a^	71 ± 9	67 ± 9	Yes	3
p.Leu555Pro		18%					ER^a^	52 ± 12	49 ± 12	Yes	3
p.Gly565Val		0%					ER^a^	0	0	Yes	1
p.His583Arg		19%					Membrane + ER	77 ± 3	80 ± 6	No	2
p.His583Tyr		19%					Membrane + ER^a^	40 ± 3	52 ± 12	Yes	3
p.Ser610Cys		0%					ER^a^	52 ± 6	56 ± 4	Yes	1

**The blue fields show detected structure features (see section “Materials and Methods” for definition of features). ER: endoplasmic reticulum; ER^*a*^: accumulation of LDLR on ER in the form of clumps; LCIM: live cell imaging microscopy; FC (loc.): LDLR plasma membrane localization determined by flow cytometry; FC (LDL int.): LDL particle internalization determined by flow cytometry; wt: wild-type; Group: three groups described in the study (see section “Discussion” for definition of groups).**

### p.Glu408Val

The variant has been described in HGMD (as disease causing), ClinVar (as likely pathogenic), LOVD (as probably affects function), and in six Czech unrelated patients but functional analysis has not been performed yet. The variant was associated with plasma membrane localization, without formation of protein clumps on ER (as seen in p.Gly565Val). No statistically significant changes of gene expression of ER resident proteins were observed. Flow cytometry revealed slightly reduced LDLR plasma membrane expression (74 ± 15% of wt) and LDL particle uptake (64 ± 11% of wt). The structural analysis showed a defect associated with the loss of a specific side chain contact.

### p.Arg416Trp

The variant has been described in HGMD (as disease causing), ClinVar (as pathogenic and likely pathogenic), LOVD (as probably affects function), and in 31 Czech patients. The variant was associated with plasma membrane localization, without formation of protein clumps on ER. No statistically significant changes of gene expression of ER resident proteins were observed. Flow cytometry showed a mild decrease of LDLR plasma membrane localization (72 ± 1% of wt) and LDL particle internalization (78 ± 15% of wt). Structural analysis showed no defect. Functional analysis of p.Arg416Trp using flow cytometry was performed also in the study (Etxebarria et al., 2015). In this study, flow cytometry showed diminished LDLR plasma membrane localization (62 ± 5% of wt), LDLR–LDL binding activity (53 ± 13% of wt), and LDL particle uptake (59 ± 1% of wt).

### p.Val429Met

The variant has been described in HGMD (as disease causing), ClinVar (as pathogenic and likely pathogenic), LOVD (as probably affects function), and in two Czech patients. The variant was associated with accumulation of the protein on ER in the form of clumps and with an increased expression of all analyzed ER resident proteins. Flow cytometry showed no LDLR on the plasma membrane and no LDL particle internalization. Structural analysis found a structural defect associated with the change of volume in buried AA.

### p.Gly478Arg

The variant has been described in HGMD (as disease causing and uncertain significance), ClinVar (as pathogenic, likely pathogenic, and uncertain significance), LOVD (as probably affects function), and in one Czech patient. On the basis of our experiments, the variant has a deleterious effect on the protein localization and function. It was associated with accumulation of the protein on ER in the form of clumps and with an increased expression of ER resident chaperones Hsp90b1, Hspa5, and Calr. Flow cytometry revealed a reduced LDLR plasma membrane expression (42 ± 4% of wt) and LDL particle internalization (64 ± 3% of wt). Structural analysis found a structural defect associated with the change of charge, polarity, and volume in buried AA.

### p.Tyr532Cys

The variant was described only in the population of Czech FH patients (1 patient). Functional analysis showed that this variant has a deleterious effect on the protein. It was associated with accumulation of the protein on ER in the form of clumps and with an increased expression of ER resident chaperones Hsp90b1 and Hspa5. Flow cytometry showed a decreased protein plasma membrane localization (71 ± 9% of wt) and LDL particle internalization (67 ± 9% of wt). Structural analysis showed a structural defect associated with the loss of specific side chain contact.

### p.Leu555Pro

The variant has been described in HGMD (as disease causing), ClinVar (as likely pathogenic), and LOVD (as probably affects function) but without performing a functional analysis so far. On the basis of our analysis, this variant has a deleterious effect on the protein localization and function. It was associated with accumulation of the protein on ER in the form of clumps and with an increased expression of ER resident chaperones Hsp90b1, Hspa5, and Calr. Flow cytometry showed a decreased protein localization on the plasma membrane (52 ± 12% of wt) and LDL particle internalization (49 ± 12% of wt). Structural analysis showed a structural defect associated with the Pro residue substitution.

### p.Gly565Val

The variant has been described in HGMD (as disease causing), ClinVar (as pathogenic and likely pathogenic), and LOVD (as probably affects function). The variant was associated with a complete retention of the protein on ER in the form of clumps and with an increased expression of all analyzed ER resident proteins. Flow cytometry revealed no LDLR plasma membrane localization and no LDL particle internalization. Structural analysis showed a structural defect associated with the change of polarity and volume in buried AA and with the Gly residue substitution.

### p.His583Arg

The variant has been described in HGMD (as disease causing), ClinVar (as likely pathogenic), LOVD (as probably affects function), and also in one Czech patients. The variant was associated with LDLR plasma membrane localization, no accumulation of the protein on ER in the form of clumps, and with no statistically significant changes of expression of ER resident proteins. Flow cytometry showed a slightly reduced LDLR plasma membrane expression and LDL particle uptake (77 ± 3% and 80 ± 6% of wt, respectively). For the p.His583Arg variant, we did not find any structural defect.

### p.His583Tyr

The variant has been described in HGMD (as disease causing and uncertain significance), ClinVar (as pathogenic, likely pathogenic, and likely benign), and LOVD (as probably affects function). This variant has a deleterious effect on the protein localization and function on the basis of our analysis. It was associated with the accumulation of the protein on ER in the form of clumps and with an increased expression of ER resident chaperones Hsp90b1 and Hspa5. Flow cytometry analysis revealed a decreased LDLR protein localization on the plasma membrane (40 ± 3% of wt) and a decreased LDL particle internalization (52 ± 12% of wt). For the p.His583Tyr variant localized on the protein surface, we did not find any defect. However, His583 is localized on the interface between the beta-propeller and the ligand binding repeat R5, where it has a close contact (3.2 Å) with His211 from R5. Most probably, the variant p.His583Tyr, resulting in loss of charge, will have a more severe effect on the protein structure than p.His583Arg.

### p.Ser610Cys

The variant has been described in HGMD (as disease causing), ClinVar (as pathogenic and likely pathogenic), LOVD (as probably affects function), and in five Czech FH patients. Functional analysis has not been performed yet. On the basis of our analysis this variant has a deleterious effect on the LDLR protein localization and function. It was associated with the accumulation of the protein on ER in the form of clumps and with an increased expression of ER resident chaperones Hsp90b1, Hspa5, and Calr. Flow cytometry revealed a decreased protein localization on the plasma membrane (52 ± 6% of wt) and a decreased LDL particle internalization (56 ± 4% of wt). Structural analysis showed a structural defect associated with the loss of a specific side chain contact and the change of polarity and volume in buried AAs.

The impact of missense variants was also analyzed using commonly used prediction programs such as MutationTaster, SIFT, and PolyPhen-2 (HumVar model). All variants were considered as disease causing and deleterious using MutationTaster and SIFT, respectively; and probably damaging using PolyPhen-2, except for p.Glu408Val and p.Val429Met that were evaluated as possibly damaging.

## Discussion

In this study, we focused on LDLR sequence variants localized in the six-bladed beta-propeller of epidermal growth factor precursor homology domain, and their effects on the LDLR protein localization, function, and on mRNA level of selected ER-resident proteins associated with ER stress and UPR. The experimental results were supplemented by the 3D protein structural analysis.

Our results show that the analyzed LDLR protein variants can be divided into three groups: (1) p.Val429Met, p.Gly478Arg, p.Gly565Val, and p.Ser610Cys. The variants are buried inside the 3D protein structure and replacing AAs change charge, polarity, and/or volume. Expression of proteins carrying the mentioned variants was associated with the ER protein accumulation in the form of clumps; significant increase in the expression of ER-resident chaperones Hsp90b1, Hspa5, and Calr; no or reduced LDLR plasma membrane localization (0%, 42 ± 4%, 0%, 52 ± 6% of wt, respectively); and no or reduced LDL particle internalization (0%, 55 ± 3%, 0%, 47 ± 4% of wt, respectively). (2) p.Glu408Val, p.Arg416Trp, and p.His583Arg. The variants are localized on the surface of 3D protein structure. Expression of the proteins was characterized by no ER protein accumulation in the form of clumps; no increased mRNA level of ER-resident chaperones; slightly reduced LDLR plasma membrane localization (74 ± 15%, 72 ± 1%, 77 ± 3% of wt, respectively) and LDL particle internalization (64 ± 11%, 78 ± 15%, 80 ± 6% of wt, respectively). (3) p.Tyr532Cys, p.Leu555Pro, and p.His583Tyr. The variants are localized on the surface of the 3D protein structure but unlike the group 2, these were associated with ER protein accumulation in the form of clumps and increased mRNA level of ER-resident chaperones. Values of LDLR plasma membrane localization are 71 ± 9%, 52 ± 12%, 40 ± 3% of wt, respectively, and LDL particle internalization 67 ± 9%, 49 ± 12%, 52 ± 12% of wt, respectively. Apparently, structural defects of these variants are more severe than in variants of the group 2.

Based on the results of testing the suitability of monitoring mRNA expression changes of various ER stress related genes, Calr, HspaA5, Hsp90b1, and Herpud1 were selected as appropriate for qRT-PCR using TaqMan probes. The CALR, HSPA5, and HSP90B1 proteins belong to the most abundant ER chaperones (Araki and Nagata, 2012). Our qRT-PCR results indicated that transcript levels of all these genes were significantly increased in cells expressing the LDLR variants p.Val429Met, p.Gly478Arg, p.Leu555Pro, p.Gly565Val, and p.Ser610Cys; in case of Hspa5 and Hsp90b1 also in p.Tyr532Cys and p.His583Tyr. For all mentioned variants, LDLR was localized predominantly on ER in the form of clumps. HERPUD1 is a component of ERAD involved in ubiquitin-dependent degradation of misfolded ER proteins (Schulze et al., 2005). We have observed increased mRNA levels of Herpud1 in the variants p.Val429Met and p.Gly565Val. From all analyzed variants, p.Val429Met and p.Gly565Val demonstrated the most serious impact on the receptor localization and function.

Analyzing LDLR localization and function was performed using live cell imaging microscopy. This methodical approach replaced our earlier methods based on classical confocal laser scanning microscopy (CLSM) using fixed cells (Pavlouskova et al., 2016). It was proven that commonly used fixation and permeabilization agents can cause extraction or re-localization of membrane bound proteins in the cell, not reflecting the *in vivo* situation (Schnell et al., 2012). We observed similar results for both fixed and live cells. Further, we performed microscopy experiments analyzing LDL particle uptake. LDL particles were conjugated with Dil (1,10-Dioctadecyl-3, 3,3′,3′-Tetramethylindocarbocyanine Perchlorate, Molecular Probes) or pHRodo-Red in CLSM and live cell imaging microscopy, respectively. Results of these analyses were also similar – all variants except p.Val429Met and p.Gly565Val were able to internalize LDL particles to a diverse extent. For a more detailed evaluation, flow cytometry was used. The cut off value for determining whether a LDLR variant is considered a functional mutant by *in vitro* studies has not been established, but based on published studies, *in vitro* LDLR activity less than 70–80% of wt protein activity (either in the LDLR plasma membrane expression or LDL particle internalization) could classify a variant as pathogenic (Benito-Vicente et al., 2015). Based on this assumption, all analyzed variants are pathogenic but with distinct effects on induction of cellular responses at the level of protein localization, accumulation, function, and expression of genes associated with ER stress.

## Data Availability Statement

The raw data supporting the conclusions of this article will be made available by the authors, without undue reservation, to any qualified researcher.

## Author Contributions

LD participated in the design of the study, performed live cell imaging microscopy, and together with JF confocal laser scanning microscopy. LN and TL performed the flow cytometry analyses. PZ performed the quantitative RT-PCR, KR 3D protein structural analyses. LT performed the selection of variants for functional and *in silico* studies. TF and LF supervised the entire project and wrote the manuscript. All authors contributed to the article and approved the submitted version.

## Conflict of Interest

The authors declare that the research was conducted in the absence of any commercial or financial relationships that could be construed as a potential conflict of interest.

## Footnotes

1 https://portal.biobase-international.com

2 https://www.ncbi.nlm.nih.gov/clinvar/

3 https://databases.lovd.nl/shared/genes/LDLR
